# Associations between respiratory illnesses and secondhand smoke exposure in flight attendants: A cross-sectional analysis of the Flight Attendant Medical Research Institute Survey

**DOI:** 10.1186/1476-069X-10-81

**Published:** 2011-09-24

**Authors:** Alexis L Beatty, Thaddeus J Haight, Rita F Redberg

**Affiliations:** 1 Department of Medicine, Division of Cardiology, University of California, San Francisco, 505 Parnassus Avenue, Box 0124, San Francisco, CA, 94143, USA; 2 Center for Family and Community Health, School of Public Health, University of California, Berkeley, 50 University Hall, MC 7360, Berkeley, CA, 94720, USA

## Abstract

**Background:**

Secondhand tobacco smoke (SHS) is associated with increased risk of respiratory illness, cancer, and cardiovascular disease. Prior to smoking bans on airlines in the late 1980s, flight attendants were exposed to a significant amount of SHS. In the present study, we examine associations between flight attendant SHS exposure and development of respiratory illnesses and cardiovascular disease.

**Methods:**

Between December 2006 and October 2010, three hundred sixty-two flight attendants completed an online questionnaire with information regarding experience as a flight attendant, medical history, smoking history, and SHS exposure. Rates of illnesses in flight attendants were compared with an age and smoking history matched population sample from NHANES 2005-2006. Logistic regression analysis was used to examine the association of reported medical conditions and pre-ban years of exposure.

**Results:**

Compared with the sample from NHANES 2005-2006, flight attendants had increased prevalence of chronic bronchitis (11.7% vs. 7.2%, p < 0.05), emphysema/COPD (3.2% vs. 0.9%, p < 0.03), and sinus problems (31.5% vs. 20.9%, p < 0.002), despite a lower prevalence of medical illnesses including high blood pressure, diabetes, high cholesterol, heart failure, cancer, and thyroid disease. Amongst flight attendants who reported never smoking over their lifetimes, there was not a significant association between years of service as a flight attendant in the pre-smoking ban era and illnesses. However, in this same group, there was a significantly increased risk of daily symptoms (vs. no symptoms) of nasal congestion, throat, or eye irritation per 10-year increase of years of service as a flight attendant prior to the smoking ban (OR 2.14, 95% CI 1.41 - 3.24).

**Conclusions:**

Flight attendants experience increased rates of respiratory illnesses compared to a population sample. The frequency of symptoms of nasal congestion, throat or eye irritation is associated with occupational SHS exposure in the pre-smoking ban era.

## Background

Secondhand tobacco smoke (SHS) is associated with increased risk of respiratory illness, cancer, and cardiovascular disease [1]. Importantly, SHS exposure is associated with increased risk of death due to cardiovascular disease of approximately 30% [1-4] and increased risk of lung cancer of 20-30%[1]. Occupational exposure to SHS is common [5,6] and the level of exposure may even exceed exposure related to home-based SHS [5]. Numerous public health measures have been instituted to limit occupational and public exposure to SHS. Evidence regarding adverse consequences of occupational exposure to SHS continues to be collected.

One population with a history of occupational SHS exposure is flight attendants. Beginning in 1988, smoking bans on flights originating from the United States (US) were gradually introduced [7]. On October 31, 1989, the US Congress voted to ban smoking on all domestic flights, and this ban went into effect on February 25^th ^1990. Smoking was banned on international flights to and from the US in 1996. Prior to the institution of these smoking bans, it was estimated that flight attendants were exposed to approximately six times the SHS of ground-based workers and fourteen times the SHS of a typical person [8].

Flight attendants reported increased physical symptoms (ocular and nasal irritation, respiratory symptoms) associated with the cabin environment [9]. However, in pre-smoking ban studies, it was not consistently clear whether these symptoms were related to the overall cabin environment or due to the effects of SHS [10]. Some individual studies suggested that symptoms of eye and nose irritation were associated with in-flight smoke exposure [11,12]. While these investigations focused on short-term symptoms related to SHS exposure in the air cabin, there are suggestions that cabin SHS exposure also has long-term effects. It has been reported that increased number of hours in a smoky cabin was associated with increased sinusitis, middle ear infections, and asthma over the lifetime of flight attendants [13]. Never-smoking flight attendants working prior to the smoking ban have been shown to have airway obstruction and impaired diffusing capacity on pulmonary function testing [14]. In addition, amongst flight attendants, the highest levels of secondhand smoke exposure were associated with increased rates of hypertension [15].

While the existing evidence suggests a relationship between flight attendant SHS exposure and development of illness, debate persists, in part due to efforts by the tobacco industry to diminish the perception of negative effects of SHS [16,17]. In the present study, we seek to evaluate associations between flight attendant secondhand smoke exposure and lifetime development of respiratory symptoms, respiratory illnesses, and cardiovascular disease.

## Methods

Between December 2006 and October 2010, 362 flight attendants were recruited via newsletters, brochures, newspaper, and internet advertisements to complete a questionnaire online (http://www.imenet.net/UCSFQuest/) or at a visit to the Flight Attendant Medical Research Institute (FAMRI) clinic at the University of California, San Francisco. Even if the flight attendants were unable to visit the FAMRI clinic, flight attendants who flew while smoking was permitted in air cabins were invited to fill out the questionnaire to study risk for diseases related to occupational secondhand smoke exposure. Questionnaire items included demographics, flight attendant occupational history, medical history, symptoms of illness, smoking history, and SHS exposure. The study was approved by the University of California, San Francisco Institutional Review Board.

### Questionnaire

Flight attendant occupational history included dates worked as a full-time and/or part-time flight attendant, cabin sections worked, and domestic/international routes. Other occupational history was also queried. Medical history was assessed by asking the respondent "Has a doctor ever diagnosed any of the following medical problems?" to a list of common cardiovascular conditions, respiratory illnesses, and cancer with response for year first diagnosed, and year of most recent worsening or event. Chest pain was assessed with the question "Do you ever experience chest pain or discomfort with exertion?" and shortness of breath was assessed with "Do you ever experience shortness of breath?" Symptoms of cough and upper respiratory symptoms were assessed by frequency of symptoms without reference to date of onset of symptoms. Personal smoking history was defined as at least 1 cigarette per day and a total of 100 cigarettes in the respondent's lifetime and further evaluated with questions regarding current and prior smoking including ages started and stopped smoking regularly, number of cigarettes smoked per day (if current smoker), and number of cigarettes smoked per day when smoking at heaviest. SHS exposure was ascertained by asking about childhood exposures, exposure in the home, exposure outside of the home, and exposure in the workplace. Assessment of smoke exposure included years of exposure and average hours per day that the participant had seen or smelled smoke in each environment.

### Comparison of FAMRI participants with NHANES sample

Participants from the FAMRI questionnaire were compared with data publicly available from participants from the US National Health and Nutrition Examination Survey (NHANES) 2005-2006 examination [18]. NHANES questionnaires were administered by NHANES staff. The majority of NHANES questions were similar to FAMRI questionnaire questions (see Additional File 1). For determination of medical history, respondents were asked "Have you ever been told by a doctor or other health professional that you had" followed by the specific medical condition. For sinus problems, the NHANES question differed from FAMRI and asked "During the past 12 months, did a doctor or other health professional tell you that you have a sinus infection?" Questions regarding symptoms of chest pain and shortness of breath differed slightly in NHANES with questions of "Have you ever had any pain or discomfort in your chest?" and "Have you had shortness of breath either when hurrying on the level or walking up a slight hill?" Smoking history in NHANES was defined as at least 100 cigarettes in the participant's lifetime. An NHANES sample was generated by restricting the population to those who were female, over the age of 18, and with a high-school education or greater. The sample was frequency-matched for age and personal smoking history (i.e., previous smoker or not and current smoking status). Comparisons between FAMRI and NHANES participants were performed using the chi-square test, and the 2-sided Fisher-exact test for less prevalent conditions (Congestive Heart Failure and Emphysema/Chronic Obstructive Pulmonary Disease).

### Definition of pre- and post-ban service as a flight attendant

Number of years of flight attendant smoke exposure was quantified by defining pre- and post- ban service. Flight attendants recorded the starting and ending month and year of service and reported whether the majority of their flight routes were domestic or international. For flight attendants who flew domestically, April 23, 1988 was defined as the date before which flight attendants had in-flight smoke exposure, since this was the date smoking bans went into effect for the majority of US domestic routes. For flight attendants who flew internationally, January 1, 1995 was used as the date before which flight attendants had in-flight smoke exposure, since this was the date of the first US airlines to ban smoking on international flights. Consideration was not made for cabin chamber worked (i.e., first class, coach), as this has not been demonstrated to have a significant effect on SHS exposure amongst flight attendants [12].

### Association of reported medical conditions with service as a flight attendant

Logistic regression analysis was used to examine the association of reported medical conditions and pre-ban years of exposure. Analysis was restricted to flight attendants who reported having never smoked (n = 235). Odds ratios (OR) of reported medical conditions (yes/no) for each decade (10-year increase) in years of pre-ban service were estimated. This duration of service was selected since most respondents were career flight attendants. OR were age-adjusted if particular medical conditions were associated with age (e.g., hypertension). OR of reported medical conditions for each per-decade increase in post-ban years and total years as a flight attendant were also estimated.

### Association of nasal congestion, throat or eye irritation (ENT) with service as a flight attendant

A subset of the 362 participants (n = 328) reported frequency of nasal congestion, throat or eye irritation (ENT symptoms). Multinomial logistic regression was used to examine the association of ENT symptoms and pre-ban years of exposure. OR of the associations between differing levels of ENT symptom frequency (daily, weekly/monthly, and less than monthly) vs. no symptoms, for every decade of pre-ban exposure, were obtained. Analysis was restricted to participants who reported ENT symptom frequency among those who reported having never smoked (n = 226 of 235). Based on availability of data where participants reported their non-occupational SHS exposure, models were adjusted for age, living with a smoker as an adult (n reporting any answer to this questionnaire item = 218 of 226), living with a smoker as a child (n = 197 of 226), and maternal exposure during pregnancy (n = 187 of 226). Additionally, analyses were performed to assess the associations of reported medical conditions and ENT symptom frequency with post-ban years and total years as a flight attendant.

Analyses were carried out with SAS version 9.1.3 and Stata version 7. Figures were created using R-software version 2.4.1.

## Results

Three hundred sixty-two flight attendants participated in the FAMRI questionnaire. Mean age amongst participants was 58.2 (interquartile range (IQR) 53-65). Amongst FAMRI participants, 116 (33.0%) had a personal history of smoking (>100 cigarettes in lifetime). Three hundred fourteen participants worked full-time in the pre-smoking ban era (median 16.9 years, IQR 9.0-23.8 years) with 8 participants working part-time in the pre-ban era (median 4.7 years, IQR 3.4-5.5 years). Two hundred eighty-two participants worked full time in the post-smoking ban era (median 14.1 years, IQR 9.2-15.2 years) with 19 participants working part-time (median 5.7 years, IQR 1.4-10.1 years). Of the participants, 76 worked exclusively in the pre-ban era, 46 worked exclusively post-ban, and 240 worked in both eras.

### Comparison of FAMRI participants with NHANES sample

Compared to an age and smoking history matched sample from NHANES 2005-2006 (Table 1), FAMRI participants had lower prevalence of high blood pressure, diabetes, high cholesterol, heart failure, cancer, thyroid disease, and chest pain. There was no statistically significant difference between NHANES and FAMRI participants for ischemic heart disease, asthma, sleep apnea, breast cancer, and shortness of breath. However, FAMRI participants had significantly increased prevalence of chronic bronchitis, emphysema/chronic obstructive pulmonary disease (COPD), and sinus problems.

**Table 1:** Prevalence of medical conditions in FAMRI questionnaire participants compared with NHANES 2005-2006 participants

	FAMRI (N = 362)	NHANES 2005-2006	
**Medical Condition**	**N (%)**	**N (%)**	**P-value**

**High Blood Pressure**	76 (22.2)	164 (47.4)	<0.001

**Diabetes**	7(2)	51 (15)	<0.001

**High Cholesterol**	110 (32.1)	148 (48.1)	<0.001

**Ischemic Heart Disease**	5 (1.5)	10 (2.9)	<0.200

**Congestive Heart Failure**	0	12 (3.5)	<0.001**

**Abnormal Heart Rhythm**	26 (7.6)	data not available	

**Asthma**	56 (16.3)	52 (15)	<0.650

**Chronic Bronchitis**	40 (11.7)	25 (7.2)	<0.050

**Emphysema/COPD**	11 (3.2)	3 (0.9)	<0.030**

**Sleep Apnea**	25 (7.3)	23 (6.7)	<0.760

**Cancer**	17 (5)	44 (12.8)	<0.001

**Breast Cancer**	7 (2)	15 (4.3)	<0.090

**Thyroid Disease**	41 (12)	77 (22.3)	<0.001

**Sinus Problems**	108 (31.5)	72 (20.9)	<0.002

**Ear Infections**	50 (14.6)	data not available	

**Chest pain**	64 (19.8)	99 (30.1)	<0.003

**Shortness of breath**	144 (44.3)	125 (38.2)	<0.115

**Nasal congestion, throat or eye irritation †**			

Daily	71 (21.6)	data not available	

Weekly	40 (12.2)		

Monthly	22 (6.7)		

< Monthly	93 (28.4)		

Never	102 (31.1)		

The National Health and Nutrition Examination Survey (NHANES) 2005-2006 sample was restricted to women above the age of 18 with at least a high school education and was age and smoking history matched with the Flight Attendant Medical Research Institute (FAMRI) participants. FAMRI participants completed questionnaires between December 2006 and October 2010. Each medical condition represents a lifetime history of diagnosis of that condition, without reference to current vs. past conditions. Chi-square test was performed for comparisons between groups, with the exception of the conditions marked with (**) which were compared using the two-sided Fisher exact test. (†) Percentages of frequency of nasal congestion, throat or eye irritation were determined using the total number of subjects responding to the question N = 328.

### Association of reported medical conditions with service as a flight attendant

Associations of medical conditions and per-decade increase in pre-smoking ban service as a flight attendant (Table 2) were determined in participants without a personal history of smoking (N = 235). Conditions associated with age (high blood pressure, high cholesterol, chronic bronchitis, chest pain, and shortness of breath) were adjusted for age. There was no significant difference in the odds of high blood pressure, high cholesterol, asthma, chronic bronchitis, sinus problems, ear infections, shortness of breath or chest pain with respect to duration of pre-smoking ban service.

**Table 2:** Association between pre-smoking ban service as a flight attendant and medical conditions

	Preban	Preban (age-adjusted)
	
Medical Condition	OR (95% CI)	OR (95% CI)
**High Blood Pressure**	1.56 (1.13-2.17)	1.1 (0.72-1.68)

**High Cholesterol**	1.34 (1.01-1.79)	1.28 (0.86-1.90)

**Asthma**	1.07 (0.76-1.52)	

**Chronic Bronchitis**	1.45 (0.97-2.17)	1.11 (0.67-1.86)

**Sinus Problems**	1.06 (0.80-1.40)	

**Ear Infections**	0.87 (0.59-1.27)	

**Chest Pain**	1.35 (0.95-1.92)	1.22 (0.76-1.97)

**Shortness of Breath**	1.22 (0.93-1.60)	1.11 (0.75-1.63)

Amongst FAMRI subjects without a personal history of smoking (N = 235), odds ratios of medical conditions per a 10-year increase in service as a flight attendant pre-smoking ban were determined using logistic regression. Conditions associated with age were age-adjusted.

Analyses did not show significant associations of reported medical conditions with per-decade increase in post-ban years or total years as a flight attendant. However, there was a non-significant trend of increased sinus problems and ear infections, respectively, with total years of service (Table 3).

**Table 3:** Association between years of service as a flight attendant with sinus problems and ear infections

Medical Condition	OR (95% CI)
**Sinus Problems**	1.27 (0.98-1.64)

**Ear Infections**	1.20 (0.86-1.69)

Amongst flight attendants without a personal history of smoking, odds ratios of reporting sinus problems and ear infections were calculated per a 10-year increase in service as a flight attendant, without regard for pre- or post-smoking ban history.

### Association of nasal congestion, throat or eye irritation (ENT) with service as a flight attendant

**Figure 1:**
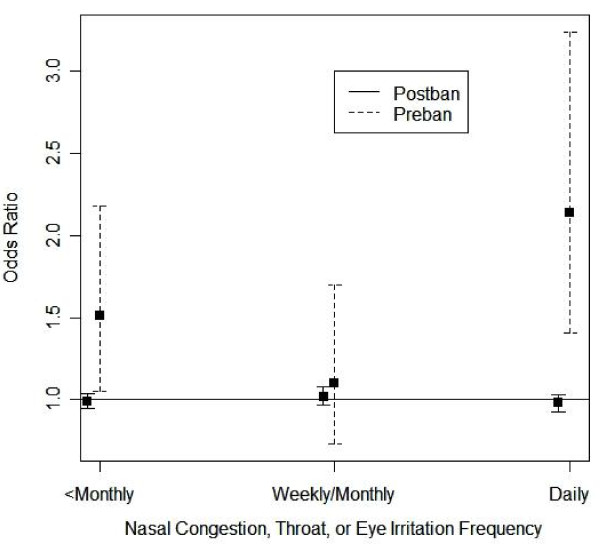
**Association of pre-ban service as a flight attendant with nasal congestion, throat or eye irritation**. Amongst flight attendants without a personal smoking history, odds ratios of reporting daily, weekly or monthly, or less than monthly symptoms of nasal congestion, throat or eye irritation (ENT symptoms) vs. never experiencing symptoms are shown per a 10-year increase in pre-smoking ban service (---) and post-smoking ban service (^___^), with point estimates represented by a solid box (■) and 95% confidence intervals represented by the region bounded by patterned lines (├─┤).

Three hundred twenty-eight of three hundred sixty-two FAMRI participants described frequency of symptoms of nasal congestion, throat or eye irritation (ENT) not related to a cold or hay fever. Of these respondents, 71 (21.6%) reported daily symptoms, 62 (18.9%) reported weekly or monthly symptoms, 93 (28.4%) reported less than monthly symptoms, and 102 (31.1%) reported never experiencing symptoms (Table 1). A similar distribution of ENT symptom frequency occurred for those participants who reported never having smoked. Analysis of symptom frequency amongst flight attendants reporting no personal history of smoking revealed a significantly higher risk of daily symptoms vs. no symptoms per decade of pre-ban service (OR 2.14, 95% CI 1.41 - 3.24)(Figure 1). There was no association between greater frequency of symptoms and years of post-ban service or total years of service. Adjustment for age, living with a smoker as an adult, and living with a smoker as a child did not attenuate this association. Adjustment for maternal exposure to smoke during pregnancy slightly attenuated the association (OR 1.65, 95% CI 0.90-3.02).

## Discussion

This questionnaire-based study of flight attendant occupational SHS exposure and medical illnesses provides several observations about the overall health of flight attendants and the relationship between illness and SHS exposure as a flight attendant. Compared with an age and smoking history matched population sample from NHANES 2005-2006, flight attendants are generally healthier than the US population with respect to most common illnesses. However, for many respiratory illnesses including chronic bronchitis, emphysema/COPD, and sinus problems, flight attendants exposed to SHS have an increased prevalence of disease. This suggests that exposures related to being a flight attendant may promote the development of respiratory illness. We did not find that years of pre-smoking ban service as a flight attendant were associated with the presence of common medical illnesses. However, frequency of symptoms of nasal congestion, throat or eye irritation was associated with years of pre-smoking ban service as a flight attendant. This association was not present for post-smoking ban years of service or total years of service, suggesting that SHS exposure during the pre-smoking ban era may explain the increased frequency of symptoms of nasal congestion, throat or eye irritation.

A previous questionnaire-based study of flight attendants by Ebbert et al [13] revealed high rates of COPD, sinusitis, allergies, bronchitis, ear infections, and asthma, but did not offer a referent comparison to the general population. Additionally, they found that sinusitis, middle ear infections, and asthma were related to hours in a smoky cabin. Our results further confirm their finding that flight attendants experience higher rates of respiratory illnesses and experience greater upper respiratory symptoms related to SHS exposure.

COPD has previously been associated with SHS exposure in several populations [14,19,20]. Amongst never-smoking flight attendants with occupational exposure to SHS, pulmonary function testing has revealed evidence of airway obstruction and impaired diffusing capacity [14]. Similarly, our results confirm prior studies reporting increased rates of bronchitis related to SHS exposure [21,22]. While several other investigations have reported an association of asthma with SHS exposure [21-23], not all have demonstrated increased rates of asthma [24]. Our findings that flight attendants experience increased risk of COPD and chronic bronchitis are consistent with these prior studies in populations with SHS exposure.

In our study, we observed fewer cancers compared to the NHANES sample, but no statistically significant difference in rates of breast cancer between the two populations. Other studies of flight attendants have yielded conflicting results regarding rates of breast cancer. Some demonstrate increased rates of breast cancer after periods of 15 or more years after initiation of service as a flight attendant, despite consideration for some potential confounders including age and reproductive status [25,26]. However, others have found that the risk of breast cancer amongst flight attendants is more associated with established risk factors for breast cancer than exposure to in flight radiation or SHS exposure [27].

Among the most common symptoms reported by flight attendants are those related to upper respiratory illness, including sinusitis, ear infections, nasal congestion, and throat or eye irritation [10]. We found a significant increase in frequency of nasal congestion, throat or eye irritation symptoms associated with duration of time as a flight attendant in the pre-smoking ban era. This finding is consistent with previous findings of increased risk of upper respiratory symptoms related to SHS exposure in general populations [28-32] and amongst flight attendants [13,24,33,34]. SHS is capable of inducing changes in the nasal mucosa by inducing biofilm formation, which may alter the nasal flora and perpetuate illness [35]. However, after the airline smoking ban took effect, a significant decrease in respirable particles and decrease in symptoms of ocular irritation amongst flight attendants was observed [11]. Given the consistent finding that symptoms of upper respiratory illness are associated with SHS exposure, further formal investigation is warranted to determine the mechanisms, chronicity, and impact of these symptoms.

Due to the nature of this questionnaire-based study, several limitations exist. Participants were recruited via newsletters, brochures, newspaper, and internet advertisements. While respondents to these advertisements were likely flight attendants, it is not possible to formally assess who responded to the ads and participated in the web-survey. 70% of those who visited the website completed the survey. The results of the study may be affected by uncontrolled selection bias of those choosing to complete the questionnaire. Self-reporting bias may overestimate the true prevalence of certain medical conditions amongst participants. Compared to the questionnaire conducted by Ebbert et al [13], the prevalence of disease within the FAMRI population is generally less frequent or similar to the prevalence of disease reported in their investigation, suggesting that bias may be slightly less than or equivalent to previous similar studies. Other sources of bias may also be present. Response rates were not 100% for each question, thus ascertainment of exposures and conditions is not complete. Data with regard to personal smoking history (i.e., age started smoking) and outside SHS exposure (e.g., years living with adult who smoked, years working in occupations where others smoked) were limited. Moreover, apart from the reported years of service aboard aircraft, the actual level of in-flight SHS exposure could not be quantified. The lack of complete responses for personal and SHS exposure limits the ability of this study to find and/or control for associations between SHS exposure and illnesses. SHS exposure determination was dependent upon responses from the questionnaire and may also be confounded by other occupational and residential exposures. With regard to comparison with other populations, the FAMRI population appears generally healthier than the NHANES population, which may be because the two populations differ with regard to numerous employment-related and socioeconomic factors. While we tried to account for some of these differences by restricting the NHANES sample to those with a high-school education or greater, we were not able to adjust for employment status or other variables with the currently available NHANES 2005-2006 data.

Additionally, the findings of increased rates of respiratory illnesses amongst flight attendants, lack of association of most illnesses with pre-smoking ban service as a flight attendant, and the non-significant trend towards increased sinus problems and ear infections related to total years of service as a flight attendant raises the possibility that some degree of upper respiratory symptoms amongst flight attendants may not necessarily be related to smoke exposure alone, but may relate to general occupational exposures related to the cabin environment. The air cabin environment includes a number of potential irritants other than tobacco smoke, including ozone [36], cleaning agents [37], volatile by-products of fuel and fuel combustion, pesticides, infectious agents, and other airborne allergens [38]. Data supporting the relationship between these agents and symptoms of respiratory illnesses are limited [38].

## Conclusions

Even though smoking has been banned from airline flights originating in the US, the effects of SHS exposure are still of interest to those flight attendants who withstood the SHS exposure and experience illness potentially related to their in-flight exposure. In addition, greater knowledge of the late effects of SHS exposure better equips health professionals to care for these individuals. Indeed, we have demonstrated that flight attendants experience increased rates of many respiratory illnesses compared to the general population, and some of the increased frequency of symptoms is related to SHS exposure. While flight attendants are no longer subject to in-flight SHS exposure, SHS exposure continues to affect individuals in other occupations and remains an area for research and advocacy.

## List of abbreviations

COPD: Chronic obstructive pulmonary disease; ENT: Symptoms of nasal congestion, throat or eye irritation; FAMRI: Flight Attendant Medical Research Institute; IQR: Interquartile range; NHANES: National Health and Nutrition Examination Survey; OR: Odds ratio; SHS: Secondhand Smoke; US: United States.

## Competing interests

RFR receives salary support from the University of California, San Francisco FAMRI grant. The other authors declare that they have no competing interests.

## Authors' contributions

ALB contributed to the design of the analysis and drafted the manuscript. TJH contributed to the design of the analysis, performed the statistical analysis, and revised the manuscript. RFR conceived of the study, and participated in its design and coordination and helped to revise the manuscript. All authors read and approved the final manuscript.

## Supplementary Material

Additional file 1 **Supplementary Table**. Comparison of question stems between the Flight Attendant Medical Research Institute Survey (FAMRI) and the National Health and Nutrition Examination Survey (NHANES) 2005-2006. Click here for file

## References

[B1] United States. Public Health Service. Office of the Surgeon General., United States. Office on Smoking and Health The health consequences of involuntary exposure to tobacco smoke: a report of the Surgeon General 2006 Rockville, MD Washington, DC: U.S. Dept. of Health and Human Services, Public Health Service for sale by the Supt. of Documents, U.S. G.P.O

[B2] Glantz SA Parmley WW Passive smoking and heart disease. Epidemiology, physiology, and biochemistry Circulation 1991 83 1 12 1984876 10.1161/01.cir.83.1.1

[B3] Taylor AE Johnson DC Kazemi H Environmental tobacco smoke and cardiovascular disease. A position paper from the Council on Cardiopulmonary and Critical Care, American Heart Association Circulation 1992 86 699 702 1638735 10.1161/01.cir.86.2.699

[B4] Whincup PH Gilg JA Emberson JR Jarvis MJ Feyerabend C Bryant A Walker M Cook DG Passive smoking and risk of coronary heart disease and stroke: prospective study with cotinine measurement BMJ 2004 329 200 205 10.1136/bmj.38146.427188.55 15229131 PMC487731

[B5] Hammond SK Exposure of U.S. workers to environmental tobacco smoke Environ Health Perspect 1999 107 Suppl 2 329 340 10.1289/ehp.99107s2329 10350518 PMC1566276

[B6] Hammond SK Sorensen G Youngstrom R Ockene JK Occupational exposure to environmental tobacco smoke JAMA 1995 274 956 960 10.1001/jama.274.12.956 7674526

[B7] Holm AL Davis RM Clearing the airways: advocacy and regulation for smoke-free airlines Tob Control 2004 13 Suppl 1 i30 36 14985614 10.1136/tc.2003.005686 PMC1766149

[B8] Repace J Flying the smoky skies: secondhand smoke exposure of flight attendants Tob Control 2004 13 Suppl 1 i8 19 14985612 10.1136/tc.2003.003111 PMC1766146

[B9] Nagda NL Koontz MD Review of studies on flight attendant health and comfort in airliner cabins Aviat Space Environ Med 2003 74 101 109 12602440

[B10] Samet JM Adverse effects of smoke exposure on the upper airway Tob Control 2004 13 Suppl 1 i57 60 14985618 10.1136/tc.2003.005454 PMC1766148

[B11] Wieslander G Lindgren T Norback D Venge P Changes in the ocular and nasal signs and symptoms of aircrews in relation to the ban on smoking on intercontinental flights Scand J Work Environ Health 2000 26 514 522 11201399 10.5271/sjweh.576

[B12] Mattson ME Boyd G Byar D Brown C Callahan JF Corle D Cullen JW Greenblatt J Haley NJ Hammond SK Lewtas J Reeves W Passive smoking on commercial airline flights JAMA 1989 261 867 872 10.1001/jama.261.6.867 2913384

[B13] Ebbert JO Croghan IT Schroeder DR Murawski J Hurt RD Association between respiratory tract diseases and secondhand smoke exposure among never smoking flight attendants: a cross-sectional survey Environ Health 2007 6 28 10.1186/1476-069X-6-28 17897468 PMC2064907

[B14] Arjomandi M Haight T Redberg R Gold WM Pulmonary function abnormalities in never-smoking flight attendants exposed to secondhand tobacco smoke in the aircraft cabin J Occup Environ Med 2009 51 639 646 10.1097/JOM.0b013e3181a7f048 19448573 PMC2722845

[B15] Ren X Hsu PY Dulbecco FL Fleischmann KE Gold WM Redberg RF Schiller NB Remote second-hand tobacco exposure in flight attendants is associated with systemic but not pulmonary hypertension Cardiol J 2008 15 338 343 18698542

[B16] Neilsen K Glantz SA A tobacco industry study of airline cabin air quality: dropping inconvenient findings Tob Control 2004 13 Suppl 1 i20 29 14985613 10.1136/tc.2003.004721 PMC1766143

[B17] Muggli ME Forster JL Hurt RD Repace JL The smoke you don't see: uncovering tobacco industry scientific strategies aimed against environmental tobacco smoke policies Am J Public Health 2001 91 1419 1423 10.2105/AJPH.91.9.1419 11527774 PMC1446797

[B18] Centers for Disease Control and Prevention (CDC). National Center for Health Statistics (NCHS). National Health and Nutrition Examination Survey Data http://www.cdc.gov/nchs/nhanes/nhanes2005-2006/nhanes05_06.htm

[B19] Eisner MD Balmes J Katz PP Trupin L Yelin EH Blanc PD Lifetime environmental tobacco smoke exposure and the risk of chronic obstructive pulmonary disease Environ Health 2005 4 7 10.1186/1476-069X-4-7 15890079 PMC1145187

[B20] Larsson ML Loit HM Meren M Polluste J Magnusson A Larsson K Lundback B Passive smoking and respiratory symptoms in the FinEsS Study Eur Respir J 2003 21 672 676 10.1183/09031936.03.00033702 12762355

[B21] Leuenberger P Schwartz J Ackermann-Liebrich U Blaser K Bolognini G Bongard JP Brandli O Braun P Bron C Brutsche M Domenighetti G Elsasser S Guldimann P Luthi JC Martin BW Medici T Perruchoud AP Radaelli A Schindler C Schoeni MH Solari G Tschopp JM Villiger B Wuthrich B Zellweger JP Zemp E Passive smoking exposure in adults and chronic respiratory symptoms (SAPALDIA Study). Swiss Study on Air Pollution and Lung Diseases in Adults, SAPALDIA Team Am J Respir Crit Care Med 1994 150 1222 1228 7952544 10.1164/ajrccm.150.5.7952544

[B22] Maziak W Ward KD Rastam S Mzayek F Eissenberg T Extent of exposure to environmental tobacco smoke (ETS) and its dose-response relation to respiratory health among adults Respir Res 2005 6 13 10.1186/1465-9921-6-13 15701169 PMC549073

[B23] Greer JR Abbey DE Burchette RJ Asthma related to occupational and ambient air pollutants in nonsmokers J Occup Med 1993 35 909 915 10.1097/00043764-199309000-00014 8229343

[B24] Whelan EA Lawson CC Grajewski B Petersen MR Pinkerton LE Ward EM Schnorr TM Prevalence of respiratory symptoms among female flight attendants and teachers Occup Environ Med 2003 60 929 934 10.1136/oem.60.12.929 14634183 PMC1740431

[B25] Pukkala E Auvinen A Wahlberg G Incidence of cancer among Finnish airline cabin attendants, 1967-92 BMJ 1995 311 649 652 7549630 10.1136/bmj.311.7006.649 PMC2551425

[B26] Rafnsson V Sulem P Tulinius H Hrafnkelsson J Breast cancer risk in airline cabin attendants: a nested case-control study in Iceland Occup Environ Med 2003 60 807 809 10.1136/oem.60.11.807 14573709 PMC1740419

[B27] Kojo K Pukkala E Auvinen A Breast cancer risk among Finnish cabin attendants: a nested case-control study Occup Environ Med 2005 62 488 493 10.1136/oem.2004.014738 15961626 PMC1741059

[B28] Bascom R Kulle T Kagey-Sobotka A Proud D Upper respiratory tract environmental tobacco smoke sensitivity Am Rev Respir Dis 1991 143 1304 1311 1710879 10.1164/ajrccm/143.6.1304

[B29] Wakefield M Trotter L Cameron M Woodward A Inglis G Hill D Association between exposure to workplace secondhand smoke and reported respiratory and sensory symptoms: cross-sectional study J Occup Environ Med 2003 45 622 627 10.1097/01.jom.0000069242.06498.86 12802215

[B30] Reh DD Lin SY Clipp SL Irani L Alberg AJ Navas-Acien A Secondhand tobacco smoke exposure and chronic rhinosinusitis: a population-based case-control study Am J Rhinol Allergy 2009 23 562 567 10.2500/ajra.2009.23.3377 19958601

[B31] Lund VJ Preziosi P Hercberg S Hamoir M Dubreuil C Pessey JJ Stoll D Zanaret M Gehanno P Yearly incidence of rhinitis, nasal bleeding, and other nasal symptoms in mature women Rhinology 2006 44 26 31 16550946

[B32] Tammemagi CM Davis RM Benninger MS Holm AL Krajenta R Secondhand smoke as a potential cause of chronic rhinosinusitis: a case-control study Arch Otolaryngol Head Neck Surg 2010 136 327 334 10.1001/archoto.2010.43 20403847

[B33] Lindgren T Andersson K Dammstrom BG Norback D Ocular, nasal, dermal and general symptoms among commercial airline crews Int Arch Occup Environ Health 2002 75 475 483 10.1007/s00420-002-0330-8 12172894

[B34] Lindgren T Norback D Health and perception of cabin air quality among Swedish commercial airline crew Indoor Air 2005 15 Suppl 10 65 72 15926946 10.1111/j.1600-0668.2005.00353.x

[B35] Goldstein-Daruech N Cope EK Zhao KQ Vukovic K Kofonow JM Doghramji L Gonzalez B Chiu AG Kennedy DW Palmer JN Leid JG Kreindler JL Cohen NA Tobacco smoke mediated induction of sinonasal microbial biofilms PLoS One 2011 6 e15700 10.1371/journal.pone.0015700 21253587 PMC3017060

[B36] Spengler JD Ludwig S Weker RA Ozone exposures during trans-continental and trans-Pacific flights Indoor Air 2004 14 Suppl 7 67 73 15330774 10.1111/j.1600-0668.2004.00275.x

[B37] Nagda NL Rector HE A critical review of reported air concentrations of organic compounds in aircraft cabins Indoor Air 2003 13 292 301 10.1034/j.1600-0668.2003.00202.x 12950593

[B38] National Research Council (U.S.). Committee on Air Quality in Passenger Cabins of Commercial Aircraft The airliner cabin environment and the health of passengers and crew 2002 Washington, D.C.: National Academy Press

