# Supplementary material for: Associations between respiratory illnesses and secondhand smoke exposure in flight attendants: A cross-sectional analysis of the Flight Attendant Medical Research Institute Survey
Source: Environ Health. 2011 Sep 24;10:81. doi: 10.1186/1476-069X-10-81 (PMC3190330; doi:10.1186/1476-069X-10-81)
Supplement: Additional file 1 — Supplementary Table. Comparison of question stems between the Flight Attendant Medical Research Institute Survey (FAMRI) and the National Health and Nutrition Examination Survey (NHANES) 2005-2006. [file 1476-069X-10-81-S1.PDF]

Additional File 1

Supplementary Table. Comparison of question stems between the Flight Attendant Medical Research Institute Survey (FAMRI) and the National Health and Nutrition Examination Survey (NHANES) 2005-2006.

Item	FAMRI	NHANES 2005-2006
**Smoking**	Have you ever smoked cigarettes regularly? (at least 1 cigarette per day and a total of 100 cigarettes in your lifetime)	Have you smoked at least 100 cigarettes in your entire life?
**Medical Conditions**	Has a doctor ever diagnosed any of the following medical problems?<br>-Year 1st Diagnosed<br>-Year of Most Recent Worsening/Event	
**High Blood Pressure**		Have you ever been told by a doctor or other health professional that you had hypertension, also called high blood pressure?
**Diabetes**		Other than during pregnancy have you ever been told by a doctor or other health professional that you have diabetes or sugar diabetes?
**High Cholesterol**		Have you ever been told by a doctor or other health professional that your blood cholesterol level was high?
**Ischemic Heart Disease**	Includes Heart Attack, Heart Bypass Surgery, Coronary angioplasty or stent.	Includes multiple responses: Has a doctor or other health professional ever told you that you had<br>-a heart attack (also called myocardial infarction)<br>-coronary heart disease
**Congestive Heart Failure**		Has a doctor or other health professional ever told you that

		you had congestive heart failure?
**Abnormal Heart Rhythm**		Not in NHANES
**Asthma**		Has a doctor or other health professional ever told you that you have asthma?
**Chronic Bronchitis**		Has a doctor or other health professional ever told you that you had chronic bronchitis?
**Emphysema/COPD**	Emphysema or COPD	Has a doctor or other health professional ever told you that you had emphysema?
**Sleep Apnea**		Have you ever been told by a doctor or other health professional that you have a sleep disorder? What was the sleep disorder? – Sleep apnea is a coded response.
**Cancer**	Lung cancer specifically queried. Any write-in responses of cancer were included.	Have you ever been told by a doctor or other health professional that you had cancer or a malignancy of any kind?
**Breast Cancer**	Ascertained from write-in responses.	What kind of cancer was it? – response available for breast cancer.
**Thyroid Disease**		Has a doctor or other health professional ever told you that you had a thyroid problem?
**Sinus Problems**		During the past 12 months, did a doctor or other health professional tell you that you have a sinus infection?
**Ear Infections**		Not in NHANES
**Chest pain**	Do you ever experience chest pain or discomfort with exertion?	Have you ever had any pain or discomfort in your chest?
**Shortness of breath**	Do you ever experience shortness of breath?	Have you had shortness of breath either when hurrying on the level or walking up a slight hill?
